# Palladium encapsulated nanofibres for scavenging ethylene from sapota fruits

**DOI:** 10.3389/fnut.2022.994813

**Published:** 2022-11-09

**Authors:** Gajanan Gundewadi, Shalini Gaur Rudra, Radha Prasanna, Tirthankar Banerjee, Sanjay Kumar Singh, Sanjay R. Dhakate, Ashish Gupta, Anjali Anand

**Affiliations:** ^1^Division of Food Science and Postharvest Technology, ICAR-Indian Agricultural Research Institute, New Delhi, India; ^2^Division of Microbiology, ICAR-Indian Agricultural Research Institute, New Delhi, India; ^3^Division of Agricultural Chemicals, ICAR-Indian Agricultural Research Institute, New Delhi, India; ^4^Division of Fruits and Horticultural Technology, ICAR-Indian Agricultural Research Institute, New Delhi, India; ^5^Advanced Materials Device and Metrology, CSIR-National Physical Laboratory, New Delhi, India; ^6^Division of Plant Physiology, ICAR-Indian Agricultural Research Institute, New Delhi, India

**Keywords:** ethylene scavenging, palladium chloride, electrospinning, nanofibre, sapota

## Abstract

Scavenging ethylene is a useful intervention during the transportation and storage of tropical climacteric fruits like sapota. Sapota (*Manilkara achras* Mill.) is a delicious tropical fruit with a very high respiration rate and poor shelf life. To prolong its post-harvest shelf life, the use of palladium chloride in electrospun nanomats was evaluated at a concentration varying from 1 to 4% levels. Encapsulation of 1–2% PdCl_2_ in nanomats increased the ethylene scavenging capacity (ESC) by 47–68%. Although, upon encapsulation, both PdCl_2_ and potassium permanganate showed significantly the same ethylene scavenging activity, the efficacy of PdCl_2_ was found better in presence of sapota fruits. The PdCl_2_ nanomats were brighter (L* > 73) in colour compared to the potassium permanganate mat. The placement of nanomats (2 cm^2^ × 9 cm^2^) in corrugated fibre board boxes in which the sapota was packed showed higher quality indices (firmness, TSS, ascorbic acid, and phenolics) along with lower PLW and respiration rate during the 8 days of storage period. Compared to control (8.35%), physiological loss in weight of 4.47% was recorded in fruits stored with ethylene scavenging nanomats. PdCl_2_ encapsulated PVA nanomats can emerge as a promising option for the retention of quality in fruits during storage and transit.

## Introduction

During storage and distribution, ethylene has a negative impact on the shelf life and quality of most fruits and vegetables. Even at very low concentrations (1), ethylene absorber compounds are used to delay the ethylene-induced ripening, softening, and deterioration of fruits and vegetables (2). Sapota’s high respiration range has been reported widely [25–35 mL CO_2_ kg^–1^ h^–1^ at 20°C (3); 16 mg CO_2_ kg^–1^ h^–1^ at 24–28°C (4)]. Its ethylene production rate varies from 10 to 100 μL C_2_H_4_ kg^–1^ h^–1^ (5, 6). This makes sapota a highly perishable fruit owing to quick ripening and senescence, and thus susceptible to post-harvest diseases. With a shelf life of only 3–5 days at 25–27°C, there is an estimated 25–30% post-harvest loss of sapota (7). These attributes make sapota an ideal candidate to test the efficacy of ethylene scavenging treatments.

Sapota (*Manilkara achras* Mill) is a tropical fruit of Sapotaceae family, commonly known as sapota or chiku in various parts of the world. This delicious, nutritious fruit with pleasant aroma is valued for its sweet and granular pulp with mellow taste (7). India, being the largest producer of sapota in the world, contributes to 10% share of global production with an annual production of 1.176 million MT (8) and exports worth 1.09 million USD (9).

Several methods have been employed to remove ethylene gas from the package or storage atmosphere commercially by using ethylene adsorption or oxidation process. The approaches to ethylene regulation can be divided into three main categories: ethylene reduction (modified atmospheric packaging through swapping the headspace gases); perforated packaging material (microperforated packaging materials); and ethylene removal by using ethylene scavenger (10, 11). Few compounds such as potassium permanganate, zeolite (activated clay), activated carbon, and palladium have been commercially exploited as ethylene scavengers for extending the shelf life of fruits and vegetables. These absorbers are generally embedded into paper bags or corrugated fibreboard boxes, sachets containing granules or nanoparticles during storage and transport of produce. Direct use of scavengers in packages or pouches poses difficulty because of toxicity and safety issues. One of the strategies to incorporate ethylene scavengers in films and mats incorporated with active ingredients for active packaging includes the use of electrospinning which draws the nanofibre either in the form of solution or molten liquid by the application of high voltage [30–50 KV (12, 13)]. The use of high voltage is necessary to eject liquid jets from the spinneret needle. These flexible electrospun nanofibre mats are highly porous and permeable which provide a high surface-to-volume ratio for interaction with compounds of interest (14).

The commercially available ethylene absorbers in the market include Neupalon (activated carbon) in Japan, Peakfresh (activated zeolite) in Australia, Evert-Fresh (activated zeolite) in USA, and Solid Ball Ethylene Absorber (activated zeolite) in India (1). Siripatrawan and Kaewklin (15) used titanium dioxide nanoparticles in the chitosan film matrix for the photocatalytic degradation of ethylene. Recently, Hernandez et al. (16) reported the use of potassium permanganate loaded sepiolite in sachets with thymol for enhancing the shelf life of tomatoes. PdCl_2_ has also generated interest amongst researchers for its property of scavenging ethylene (17–19). To override toxicity issues, PdCl_2_ is being used by impregnation on zeolites, activated carbon, etc. Activated carbon-embedded palladium (1%) could remove the ethylene from the storage atmosphere of tomato fruits, extending their shelf life (20). Zeolite-impregnated palladium showed a significant effect in terms of ethylene adsorption and could control the ripening and senescence of climacteric fruits, such as avocado and banana (19, 21). In another study by Cao et al. (22), the senescence and yellowing of broccoli florets were significantly reduced by the use of acidified activated carbon impregnated with palladium chloride and copper sulfate.

However, ethylene scavenging potential and fruit quality enhancing property of PdCl_2_ were less investigated, particularly, through impregnation in nanofibre mats and use in packaging material. The present investigation focused on developing a suitable technology using palladium chloride encapsulated nanomats for improving the post-harvest quality of sapota, a popular fruit in tropical countries.

## Materials and methods

### Experimental material and chemicals

Poly vinyl alcohol (PVA, M.W. 85,000 to 1,24,000) base polymer was purchased from CDH, New Delhi, India for use as a base material for forming the nanofibres. Palladium (II) chloride (PdCl_2,_ M.W. 177.33; palladium content 59–60%) was purchased from Merck and potassium permanganate (KMnO_4_, M.W. 158.03 and purity 99.5%) was purchased from SRL Chemicals, Mumbai, India. All solutions were prepared using Ultra-pure Grade-II water obtained from a Milli-Q filtration system.

### Development and characterisation of electrospun nanofibre

Electrospinning machine (ESPIN-NANO, Physics Instruments CO (PICO), Chennai, India equipped with a variable electrical voltage 10–23 kV power supply was used to prepare nanofibre mats. The composition of the PVA nanofibre mat was optimised for electrospinning conditions: voltage 15 kV, distance 20 cm and flow rate 0.2 mL/h, PVA solution of 10% and essential oils (thyme, betel leaf), β-cyclodextrin (1%) for the formation of smooth and uniform nanofibres (200–300 nm) free of beads (8). Further, ethylene scavenging capacity was induced in composite nanofibres 1–4 by the incorporation of palladium (II) chloride (1, 2, 3, and 4%, respectively) to form molecular inclusion complexes with volatile essential oils. The composition of different composite nanofibres is shown in Table 1. A single dose KMnO_4_ (4%)-based nanofibre (CNF5) was also developed as a check (keeping other parameters the same as PdCl_2–_based nanofibres) for comparing the efficacy of PdCl_2_.

**TABLE 1:** Composition of electrospun composite nanofibers.

S. no.*	Thyme essential oil (ppm)	Betel leaf essential oil (ppm)	Palladium chloride (%)	β-cyclodextrin (%)	Potassium permanganate (%)
CNF1	100	100	1	1	–
CNF2	150	150	2	1	–
CNF3	200	200	3	1	–
CNF4	150	150	4	1	–
CNF5	150	150	0	1	4

*Composition: PVA/PdCl_2_/EO/β-CD.

### Characterisation of nanofibres

Scanning electron microscope (VEGA-3-SBH, TESCAN, Czech) was used to study the surface morphology of the developed PVA/PdCl_2_/essential oil/β-cyclodextrin nanofibres (8).

#### X-ray diffraction

The palladium (II) chloride-loaded nanofibres were characterised using X-ray diffraction (XRD, D8 Advance, Bruker, Japan) with a diffraction angle between 5° and 50° for identifying the structural properties of loaded compounds. The metal components were identified with the help of their retention time and use of standard library: Joint Committee on Powder Diffraction Standards.

#### Ethylene scavenging capacity

Gas chromatograph (Bruker 450, Bruker Inc., Billerica, MA, USA) was used to determine the ethylene absorption capacity of developed nanofibres. The experiment was conducted in test tubes capped with a rubber gasket. Nanofibres (5 cm^2^) prepared with the same percentage of PdCl_2_ were compared with equivalent quantities in the aqueous solution (1 mL). Prepared nanofibres (control, CNF1, CNF2, CNF3, CNF4, and CNF5) were attached to the inside wall of test tubes (30 mL) exactly in centre and capped hermetically with a rubber gasket. Similarly, the tubes with the PdCl_2_ in varied aqueous solutions of 0–4% (1 mL) were prepared. Ethylene gas (50.8 ppm) was injected into test tubes using a gas injection syringe and the cap was sealed with paraffin film. After 4 h, gas was sampled from tubes with help of a micro syringe and injected into GC-MS to determine the remaining ethylene in each test tube.

#### Colour

The colour of developed nanofibre mats was determined by Hunter Lab spectrophotometer (MiniScan EZ, Reston, USA) using the Hunter colour parameters L*, a*, b*, Chroma (C), and hue angle (h). The L* indicates lightness co-efficient and ranges from 0 to 100 (black to white), and a* and b* indicate the colour ranges from green to red and blue to yellow from negative to positive on the horizontal and vertical axes, respectively. From these L*, a*, and b* values, the total colour change (ΔE), Hue, and chroma were calculated using the following formula. At least five measurements were performed at different points of the fibre mat (23).


(1) $$\mathrm{Total}\mathrm{colour}\mathrm{change}\left(\Delta E\right)=\sqrt{{\left(L-L_{o}\right)}^{2}+{\left(a-a_{o}\right)}^{2}+{\left(b-b_{o}\right)}^{2}}$$

where, *L_*o*_, a_*o*_*, and *b*_*o*_ are initial values and *L*, *a*, and *b* are final values.


$$Chroma=\sqrt{a^{2}+b^{2}}$$

$$Hue\left(h\right)=tan^{-1}\frac{b}{a}$$

## Effect of in-pack composite nanofibre on sapota fruits

The effect of in-pack placement of developed CNF on post-harvest quality (physical, physiological and biochemical properties) of Sapota cv. Kalipatti was evaluated during storage at 26 ± 2°C for 8 days. Composite nanofibre (CNF1 to CNF5) mats of 18 cm^2^ area in each treatment were cut into two pieces (9 cm^2^ each) and attached to a corrugated fibreboard box (CFB: 25 cm^3^ × 25 cm^3^ × 15 cm^3^, fruit weight ∼560 g/box). The CNF patches were pasted under both flaps of the lid of the box. Aeration holes (0.5 cm^2^) were made on all four sides of the box. Fruits were evaluated for their quality and shelf life on alternative days. All parameters were analysed in triplicates. One fruit was randomly sampled from each of the three boxes.

### Fruit firmness

The firmness of sapota fruits was measured at three different points along their circumference, as well as the force required to puncture the fruits, using a texture analyser (TA-XT plus, Stable Microsystems, UK) and a 5 kg load cell. About 2 mm diameter probe was used with a test speed of 0.5 mm s^–1^ and penetration depth of 5 mm (24). The firmness was defined in terms of maximum force (N) during the compression. The first peak in the force deformation curve was taken as the firmness of the fruit. Internal flesh toughness (N.s) was determined by computing the area between the first and last peaks of the force–time curve.

### Physiological loss in weight

Individual fruits were numbered in each treatment to record PLW. The weight was recorded using an electronic balance (AND, FX-2000, Japan) on alternative days during storage. The results reported are based on observations of 10 fruits and cumulative PLW was calculated in percentage using the following formula (25).


(2) $$\text{PLW}\left(\%\right)=\frac{\left(\mathrm{Initial}\mathrm{weight}-\mathrm{Weight}\mathrm{on}n^{th}\mathrm{day}\right)}{\mathrm{Initial}\mathrm{weight}}\times 100$$

### Respiration rate

The respiration rate of sapota fruits was estimated using respiratory gas analyser (PBI Dansensor, Denmark) under ambient conditions. Fruits from different treatments were kept in a hermetically sealed 1,000 mL container for 2 h at 26°C. The headspace gas was sampled by piercing the syringe attached to rubber septa fixed on the lid of the container for recording the O_2_ and CO_2_ values. The respiration rate was determined using the formula (26):


(3) $$\mathrm{Respiration}\mathrm{Rate}\left(\mathrm{mg}\mathrm{of}{\text{CO}}_{2}/\text{kg}/h\right)=\frac{\%{\text{CO}}_{2}\times \mathrm{Head}\mathrm{Space}}{\mathrm{Fruit}\mathrm{weight}\left(\text{g}\right)\times \mathrm{Enclosure}\mathrm{time}\left(\text{h}\right)\times 100}$$

### Ethylene evolution rate

#### Calibration of the gas chromatograph

A Hewlett Packard (H.P.) gas chromatograph (5890 Series II) equipped with a flame ionisation detector (FID), Porapak-N 80/100 mesh packed stainless steel column and a HP integrator was used for the determination of ethylene. The temperature of injector, column and detector was adjusted to 110, 60, and 275°C and the flow rate of nitrogen, hydrogen, and air was maintained as 30, 30, and 300 mL/min, respectively. Some amount of ethylene was collected into a fraction collector from standard calibration gas of ethylene (EDT research, London, UK). One milliliter of standard ethylene was drawn from a fraction collector using a Hamilton gas-tight micro syringe and injected into the G.C. The integrator was calibrated by recording the retention time and peak area of the standard ethylene gas.

#### Sample preparation

Three fruits from each replication were marked for the determination of ethylene evolution. Once the chromatograph was standardised and calibrated, 1 mL gas sample was drawn through the sub-seal septum with the help of a gas-tight micro syringe after a specified time of trapping the fruit (3 h). The sample was injected into the GC and the concentration of evolved ethylene (ppm) within the time interval was recorded from the integrator. The rate of ethylene evolution was expressed as μL kg^–1^ h^–1^ (27).


(4) $$\mathrm{Ethylene}\mathrm{evolution}\mathrm{rate}\left(\mu \text{L}{\text{C}}_{2}{\text{H}}_{4}/\mathrm{kg}/h\right)=\frac{C_{2}H_{4}\left(\mu L/L\right)\times \mathrm{Volume}\left(\mathrm{mL}\right)}{\text{weight}\left(\text{kg}\right)\times \mathrm{time}\left(h\right)}$$

### Physico-chemical parameters

Sapota fruits were peeled, pulped, and homogenised for the determination of total soluble solids by using a hand refractometer (Atago, Japan). The values were corrected for 20°C and expressed as °Brix. Titratable acidity (TA) and ascorbic acid content of sapota fruit were estimated using the titration method (28). Total phenols and total flavonoids were determined for 80% ethanolic extracts using spectrophotometric assay (29).

### Acceptability score

The acceptability score of fruits was evaluated by a semi-trained panel of 10 judges (four men and six women, aged between 25 and 37 years). All the panellists were trained and had sound expertise in the sensory evaluation of foods. The samples were evaluated on a hedonic scale of 1–9. Fruits were placed on a small white coloured plate with a three-digit code on the side and served to judges. The judges evaluated the intensity of each descriptor by assigning a categorical nine-point hedonic scale. The sample order for each panellist was randomised (30).

### Decay index

The decay incidence (in percentage) was determined based on the number of fruits which showed signs of decay over the initial number of fruits. The cumulative number of decayed fruits during storage and ripening was recorded and expressed as a percentage.


(5) $$\mathrm{Decay}\mathrm{Index}\left(\%\right)=\frac{\mathrm{Number}\mathrm{of}\mathrm{infected}\mathrm{fruits}}{\mathrm{Total}\mathrm{number}\mathrm{of}\mathrm{fruits}}\times 100$$

## Statistical analysis

The experiment was carried out in a completely randomised design with three replications. The results were analysed using ANOVA and the treatment means were compared using DMRT (Duncan’s multiple range test) values at a significance level *p* < 0.05. All the statistical analyses were conducted using SPSS Statistics 17.1 (IBM, New York, USA).

## Results and discussion

During our previous work on formulating essential oil encapsulated PVA nanomats to counter the incidence of anthracnose in sapota, the need to arm the nanomat with ethylene scavenger was realised. Although potassium permanganate is commonly used for ethylene scavenging, palladium chloride, a relatively less exploited salt was used to compare the efficacy *in vivo* form for a high respiring crop like sapota. Since the study targets the packaging requirements of sapota, the experiments were conducted at an average room temperature of the growing areas (26 ± 2°C) to simulate the transit conditions prevalent in the cultivation areas of Gujarat and Karnataka in India.

### Scanning electron microscopy

**FIGURE 1:**
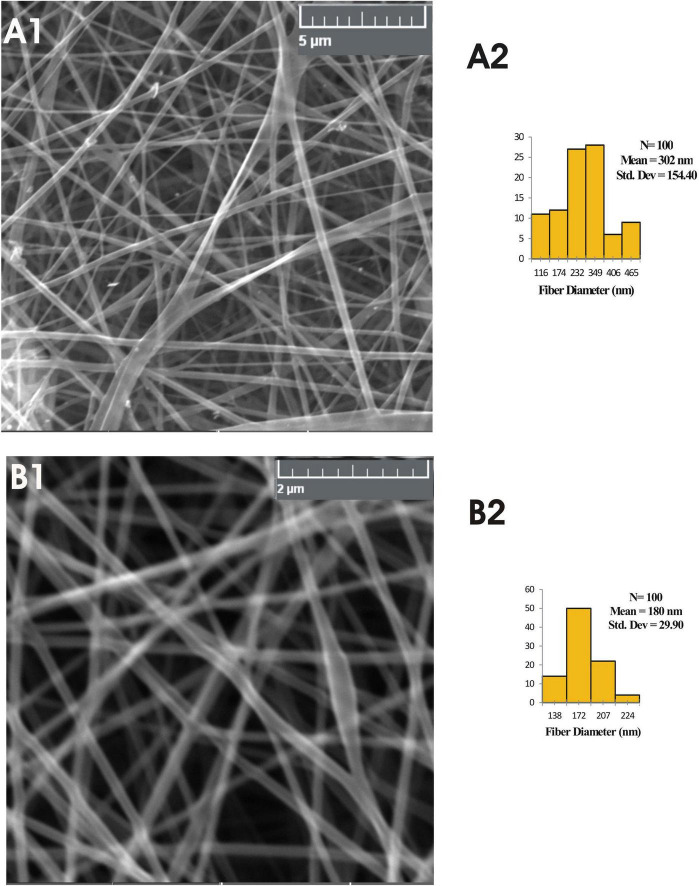
Scanning electron microscopic images **(A1,B1)** and diameter distribution histograms **(A2,B2)** of the composite nanofiber mats 4 and 5.

The typical SEM images and fibre diameter histograms of selected encapsulated PVA nanomats are shown in Figure 1. The mean diameter of the CNF1, CNF2, CNF3, CNF4, and CNF5 nanofibres showed an average diameter of the fibre as 243, 256, 233, 302, and 180 nm, respectively. These are in the range of the fibres prepared with essential oils in our previous study (8). With an increase in PdCl_2_ concentration from 3 to 4%, the fibre diameter also increased from 233 to 302 nm. The KMnO_4_-based fibre (CNF5) showed the least diameter which may be attributed to its perfect dispersion and distribution in nanofibre mats. Our results agree well with the already reported results by Wen et al. (31, 32) who reported an average size of PVA/CEO/β-CD between 300 and 410 nm and polyvinylpyrrolidone fibres between 230–428 nm in electrospun fibre, respectively.

### X-ray diffraction

The X-ray diffraction patterns of developed composite nanofibre. XRD is widely used to determine the presence of incorporated compounds by identifying crystallinity peaks. PVA is a semi-crystalline polymer and the XRD of PVA showed broad diffraction at around 2θ∼19.6° and 2θ∼22.5° (33). PdCl_2_ being a metallic element, its presence in mats was confirmed through broad diffraction at around 2θ∼12° and 2θ∼42.5°. The aluminium diffraction was noticed at around 2θ∼38.5° and 2θ∼45° as it was used as a collector blasé plate during the electrospinning process (33).

### The ethylene scavenging capacity of palladium (II) chloride

The observations on ethylene scavenging capacity (ESC) of free PdCl_2_ and encapsulated PdCl_2_ are shown in Table 2. The scavenging potential increased with the concentration of free PdCl_2_. ESC showed a linear increase from 1 to 3%; however, at the 4% level, the ESC increased drastically. ESC (800–814 ppm) for free salts of PdCl_2_ and KMnO_4_ at 4% concentration was found equivalent.

**TABLE 2:** Ethylene scavenging capacity of free and palladium (II) chloride encapsulated composite nanofibres.

Free PdCl_2_		PdCl_2_ encapsulated composite nanofibres	
Solution	Mean values (ppm)	CNF	Mean values (ppm)
Control	0.00 ± 0.00	Control	0.00 ± 0.00
PdCl_2_-1%	285.60 ± 33.67^a,1^	CNF1	420.00 ± 35.38^a,2^
PdCl_2_-2%	322.20 ± 34.89^b,1^	CNF2	541.67 ± 33.11^b,2^
PdCl_2_-3%	487.80 ± 33.78^c,1^	CNF3	615.00 ± 32.11^c,2^
PdCl_2_-4%	813.60 ± 0.00^d,1^	CNF4	842.22 ± 16.95^d,2^
KMnO_4_-4%	811.78 ± 0.00^d,1^	CNF5	822.22 ± 22.04^d,1^

Means with different uppercase letters indicate significant difference within a column at *p* < 0.05 and different uppercase numbers indicate significant difference across rows (Duncan’s Multiple Range Test); CNF1: 1%; CNF2: 2%; CNF 3: 3%; CNF4: 4% PdCl_2_; and CNF 5: 4% KMnO_4_.

Similarly, in case of nanofibre, the scavenging potential was the highest for 4% PdCl_2_ containing CNF (842.22 ppm: Table 2). Higher ESC was recorded when PdCl_2_ was present in nano-encapsulated form at all concentrations studied. Encapsulated PdCl_2_ showed 47.16, 68.11, 26.08, and 3.52% higher ESC over soluble salts at 1, 2, 3, and 4% concentrations, respectively. Encapsulated KMnO_4_ also recorded 1.29% higher ESC compared to its soluble counterpart. This is in accordance with the reported benefits for use of electrospinning as it generates a highly porous interactive surface for active encapsulates to react with the package environment (34). Also, there was no significant difference between the PdCl_2_ encapsulated (CNF4) nanofibres and KMnO_4_ nanofibre (CNF5), as both the treatments showed ESC of 842.22 and 822.22 ppm, respectively.

### Colour of nanofibre

The colour of the nanomat might have implications in terms of the acceptability of the technique and perception of purity and efficacy. The increased dose of palladium chloride in composite nanofibres led to an increase in Chroma, hue, and total colour. PVA nanofibre was the brightest (L* = 96.5) nanofibre among all. As the dose of PdCl_2_ increased, the L* value decreased, with the least value recorded for CNF5 (L* = 68.20). CNF5 was the darkest among all the nanofibres (a* = 2.20) followed by CNF4 (a* = 1.92) and the least colouration was observed in only PVA nanofibre (a* = 1.92). The chroma value for PVA was 0.291 which increased from 5.06 for CNF1 to 11.92 for CNF5. Hue value also increased from 1.36 in CNF1 to 1.38 in case of CNF5. The total colour change was least noticed in case of CNF1 (116.11) as compared to highest in CNF5 (949.16). Akinalan Balik et al. (35) in their study on electrospun pectin-based films also reported that for two pectin interlayers’ generated multilayer films, an observer could notice different colours (ΔE*_ab_ ≥ 5) due to the difference in values of b*. Similarly, Fayemi (36) has mentioned a change in colour of polystyrene-based electrospun fibres loaded with palladium chloride (PdCl_4_^2–^).

## Effect of composite nanofibre on sapota quality during storage

### Physiological loss in weight

**FIGURE 2:**
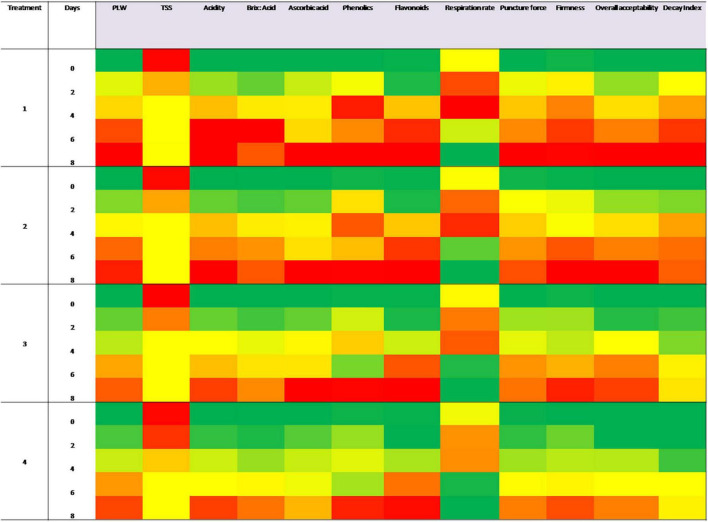
Heatmap for physicochemical quality parameters of sapota during storage.

The presence of CNF in the package of sapota fruits led to a substantial decrease in the physiological loss in weight (%) during storage at 26 ± 2°C (Table 3). This could be attributed to higher integrity of fruit tissues, owing to delay in changes leading to senescence by restricting respiration rate. Similar findings of a decrease in senescence-associated changes have been reported by Cao et al. (19) who used activated carbon–PdCl_2_ and copper sulphate matrix as ethylene scavengers for broccoli. Similarly, Kumar and Thakur (37) have reported lower PLW for pear stored in LDPE film bags containing 15% KMnO_4_ and 10% iron powder sachets. A progressive loss in the weight of fruit was observed in all treatments during the storage period of up to 8 days. The weight loss of control treatment on day 2 was 3.85% which continued to increase to 17.72% on day 8 recording mean weight loss of 8.35%. This shows a close similarity with the report by Khanvilkar (38) for 13.44% PLW on day 6 for sapota cv. Kalipatti. In contrast, with the CNF4 treatment, 1.15% weight loss was registered on day 2, which continued to increase to 11.08% on day 8, registering mean weight loss of 4.47% for the entire storage period. PLW decreased as a concentration of scavengers increased in nanomats. No significant difference was observed in case of control and CNF1 treatments as lower dose of PdCl_2_ proved to be non-significant in terms of controlling the weight loss as compared to higher dose treatments. On day 6 of storage, 1.78- and 1.56-fold higher PLW was recorded for control and CNF1 fruits compared to CNF 5. Weight loss on day 8 of storage in case of CNF1, CNF2, and CNF3 was found to be 16.17, 12.94, and 12.05%, respectively. Results concluded that among all the treatments CNF4 and CNF5 performed significantly better in terms of enhancing the shelf life of sapota fruit by delaying the weight loss in fruit. Generally, for subtropical climate, PLW exceeding 10% marks the end of storage life as the fruits become unmarketable due to visible signs of shrivelling. Our research clearly shows that with CNF4 treatment, fruits can be marketed for up to 6–7 days under ambient conditions, compared to 4 days in the control group. As can be seen in the heat map (Figure 2), the performance of CNF4 was better than all other treatments during storage with highest green shaded portion. Our treatment was as effective as that of the other researchers (39). Also, it was observed that CNF4 fruits recorded lower PLW over CNF5 fruits, indicating the better performance of PdCl_2_ over KMnO_4_ in a real situation. This could be attributed to higher reactivity for PdCl_2_ fibres in a moderate humid environment as that of the package, compared to KMnO_4_. As Hoechst–Wacker reaction is responsible for ethylene scavenging, the role of in-package moisture due to respiration is also a consideration. Recently, Shenoy et al. (40) investigated the efficacy of TiO_2_ modified with Pd at three relative humidity levels (RH: 0, 50, and 100%) and found that palladium, when deposited on TiO_2_, acted better at 0 RH and as an ethylene adsorber at higher RH, demonstrating ethylene desorption after 3 weeks. Smith et al. (18) have also reported higher efficacy of ethylene scavenging by Pd over KMnO_4_ when used in lower quantities at higher humidities. The fact that the ESC experiments in test tubes were carried out at ∼67% RH while in-pack humidity was close to 95% may also explain better performance of PdCl_2_ over KMnO_4_. Terry et al. (41) have also reported 6-fold higher scavenging activity of palladium chloride over potassium permanganate (∼ 4,162 μL g^–1^) under approximately 100% RH. They observed that rather than blocking the perception of ethylene, the Pd-promoted material effectively removes ethylene rapidly from an environment, and thus there is less risk that unwanted side effects occur. Further, Tzeng et al. (42) used zeolites to encapsulate palladium to achieve a highly active catalyst with stability for improving the shelf life of bananas. However, encapsulation in PVA nanofibres seems to have reactive action, though further studies are needed to validate this a reacting action rather than scavenging action.

**TABLE 3:** Effect of in-pack composite nanofibre placement on physicochemical quality of sapota fruits during storage at 26 ± 2°C.

Parameter	Treatment	Storage period (days)				
		0	2	4	6	8
PLW	Control	0.00 ± 0.00^a^	3.85 ± 0.03^e^	6.43 ± 0.12^e^	13.77 ± 0.90^e^	17.72 ± 0.45^d^
	CNF1	0.00 ± 0.00^a^	2.23 ± 0.09^d^	4.81 ± 0.25^d^	12.28 ± 0.21^d^	16.17 ± 0.30^d^
	CNF2	0.00 ± 0.00^a^	1.72 ± 0.02^c^	3.18 ± 0.47^b^	9.05 ± 0.19^bc^	12.94 ± 0.69^c^
	CNF3	0.00 ± 0.00^a^	1.28 ± 0.03^b^	3.43 ± 0.25^b^	9.76 ± 0.10^c^	12.05 ± 0.30^c^
	CNF4	0.00 ± 0.00^a^	1.15 ± 0.04^a^	2.39 ± 0.16^a^	7.72 ± 0.99^a^	11.08 ± 0.81^a^
	CNF5	0.00 ± 0.00^a^	1.63 ± 0.05^c^	4.01 ± 0.05^c^	8.79 ± 0.37^a^	12.53 ± 1.51^ab^
TSS	Control	16.27 ± 0.12^a^	19.07 ± 0.45^d^	22.67 ± 0.14^d^	25.10 ± 0.08^b^	20.32 ± 0.08^a^
	CNF1	16.33 ± 0.12^a^	18.90 ± 0.16^d^	22.10 ± 0.68^d^	25.00 ± 0.14^b^	20.33 ± 0.47^a^
	CNF2	16.20 ± 0.08^a^	18.23 ± 0.37^b^	21.07 ± 0.78^c^	23.20 ± 0.45^a^	20.67 ± 0.47^ab^
	CNF3	16.30 ± 0.08^a^	17.03 ± 0.09^c^	19.50 ± 0.16^b^	23.50 ± 0.57^s^	22.33 ± 0.17^d^
	CNF4	16.27 ± 0.05^a^	16.27 ± 0.17^a^	17.00 ± 0.08^a^	23.10 ± 0.14^a^	21.53 ± 0.33^c^
	CNF5	16.27 ± 0.12^a^	16.20 ± 0.08^a^	17.10 ± 0.16^a^	23.77 ± 0.29^a^	21.23 ± 0.12^bc^
Acidity (%)	Control	0.20 ± 0.02^a^	0.19 ± 0.00^a^	0.13 ± 0.00^a^	0.09 ± 0.03^a^	0.06 ± 0.00^a^
	CNF1	0.20 ± 0.02^a^	0.19 ± 0.00^a^	0.13 ± 0.00^a^	0.11 ± 0.03^bc^	0.06 ± 0.00^a^
	CNF2	0.20 ± 0.02^a^	0.19 ± 0.00^a^	0.15 ± 0.03^b^	0.13 ± 0.00^cd^	0.09 ± 0.03^b^
	CNF3	0.20 ± 0.02^a^	0.19 ± 0.00^a^	0.17 ± 0.03^c^	0.15 ± 0.03^d^	0.09 ± 0.03^b^
	CNF4	0.20 ± 0.02^a^	0.20 ± 0.02^b^	0.17 ± 0.03^c^	0.15 ± 0.03^d^	0.13 ± 0.00^c^
	CNF5	0.20 ± 0.02^a^	0.20 ± 0.02^b^	0.17 ± 0.03^c^	0.15 ± 0.03^d^	0.13 ± 0.00^c^
Ascorbic acid (mg/100g)	Control	18.00 ± 0.00^a^	15.33 ± 0.47^a^	13.00 ± 0.00^a^	9.78 ± 0.00^a^	6.80 ± 0.01^a^
	CNF1	18.00 ± 0.00^a^	17.67 ± 1.25^b^	13.33 ± 0.48^a^	9.89 ± 0.46^ab^	6.90 ± 0.00^a^
	CNF2	18.00 ± 0.00^a^	17.67 ± 0.47^b^	13.67 ± 0.46^a^	10.45 ± 0.47^b^	7.85 ± 0.00^b^
	CNF3	18.00 ± 0.00^a^	18.00 ± 0.00^b^	15.33 ± 0.47^b^	14.33 ± 0.47^c^	10.00 ± 0.00^c^
	CNF4	18.00 ± 0.00^a^	18.33 ± 0.47^bc^	15.67 ± 0.47^b^	15.33 ± 0.47^d^	12.67 ± 0.47^d^
	CNF5	18.00 ± 0.00^a^	18.33 ± 0.47^bc^	15.67 ± 0.47^b^	15.00 ± 0.00^d^	12.33 ± 0.47^d^
Total phenolics content (mg GAE/100 g)	Control	25.78 ± 0.04^a^	19.29 ± 0.57^a^	14.08 ± 0.19^a^	7.94 ± 0.26^a^	5.56 ± 0.03^a^
	CNF1	25.79 ± 0.05^a^	20.08 ± 0.20^b^	14.63 ± 0.18^b^	8.67 ± 0.26^b^	5.56 ± 0.03^a^
	CNF2	26.25 ± 0.02^bc^	20.80 ± 0.07^b^	15.36 ± 0.l3^c^	11.08 ± 0.16^c^	6.00 ± 0.03^b^
	CNF3	25.94 ± 0.05^abc^	22.09 ± 0.02^c^	18.96 ± 0.16^d^	13.53 ± 0.29^d^	9.35 ± 0.03^c^
	CNF4	25.88 ± 0.00^ab^	24.05 ± 0.02^d^	19.88 ± 0.17^e^	17.33 ± 0.18^e^	11.61 ± 0.03^d^
	CNF5	26.34 ± 0.00^c^	23.70 ± 0.06^d^	18.92 ± 0.12^d^	16.20 ± 0.18^f^	9.90 ± 0.03^c^
Total Flavonoids (mg/100 g in quercetin)	Control	7.83 ± 0.00^a^	7.28 ± 0.03^a^	4.47 ± 0.02^a^	3.05 ± 0.02^a^	2.60 ± 0.02^a^
	CNF1	7.83 ± 0.00^a^	7.31 ± 0.04^a^	4.50 ± 0.01^a^	3.16 ± 0.01^b^	2.66 ± 0.01^a^
	CNF2	7.83 ± 0.00^a^	7.32 ± 0.03^a^	5.53 ± 0.02^b^	3.45 ± 0.02^c^	2.66 ± 0.03^a^
	CNF3	7.83 ± 0.00^a^	7.57 ± 0.02^b^	5.88 ± 0.03^c^	3.72 ± 0.03^d^	2.78 ± 0.02^b^
	CNF4	7.83 ± 0.00^a^	7.81 ± 0.02^c^	6.33 ± 0.02^e^	4.56 ± 0.02^f^	3.12 ± 0.02^d^
	CNF5	7.83 ± 0.00^a^	7.53 ± 0.02^b^	6.05 ± 0.02^d^	4.00 ± 0.03^e^	3.00 ± 0.02^c^

All data are means of three replicate samples. Means with different uppercase letters within a column are significantly different at *p* < 0.05 (Duncan’s Multiple Range Test); CNF1: 1%; CNF2: 2%; CNF 3: 3%; CNF4: 4% PdCl_2_; and CNF 5: 4% KMnO_4_.

### Respiration rate

**FIGURE 3:**
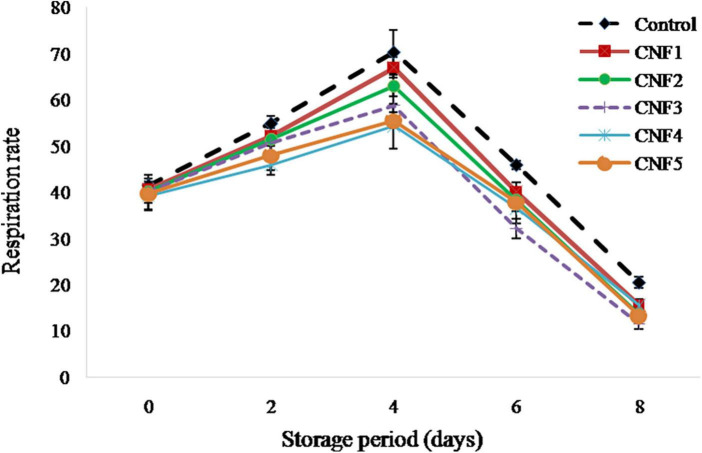
Respiration rate of sapota fruits stored in corrugated fibre board boxes with CNF during storage at 26 ± 2°C.

The respiration rate of sapota fruit subjected to different treatments is shown in Figure 3. The respiration rate of fresh sapota fruits in case of control day was recorded as 41.56 mL CO_2_ kg^–1^ h^–1^ which increased to 70.39 mL CO_2_ kg^–1^h^–1^ on day 4 of storage. Highest respiration rate values on day 4 of storage for the fruits arranged in descending order, are as follows: control (70.39 mL CO_2_ kg^–1^ h^–1^) > CNF1 (67.02 mL CO_2_ kg^–1^ h^–1^) > CNF2 (63.05 mL CO_2_ kg^–1^ h^–1^) > CNF3 (58.80 mL CO_2_ kg^–1^ h^–1^) > CNF4 (54.34 mL CO_2_ kg^–1^ h^–1^) > CNF5 (55.57 mL CO_2_ kg^–1^ h^–1^). Bhutia et al. (43) also reported that the respiration rate of Sapota cv. Kalipatti along with KMnO_4_ ethylene absorbent (59 mL CO_2_ kg^–1^ h^–1^) was significantly less as compared to control (72 mL CO_2_ kg^–1^ h^–1^) on day 3 of storage.

As sapota is a climacteric fruit, its physiological maturity results showed a significant increase in the respiration rate until day 4 of storage followed by a decrease in the respiration rate till the end of the storage period. The higher respiration rate in case of control sapota fruits is mainly due to higher ethylene production, which is known to stimulate enhanced respiration rate. Considering the cumulative ethylene produced (498.39 ppm) by sapota during storage, theoretically, CNF3 with a scavenging capacity of 615.00 ppm seemed adequate in terms of its performance. In practice, however, the respiration rate was found significantly less in case of CNF4 treatment of sapota as compared to CNFs 1–3 (Figure 2). Our findings are in range with those of Moo-Huchin et al. (44) for sapodilla fruits treated with 1-MCP at 1 μL/L concentration.

### Fruit firmness and puncture force

**FIGURE 4:**
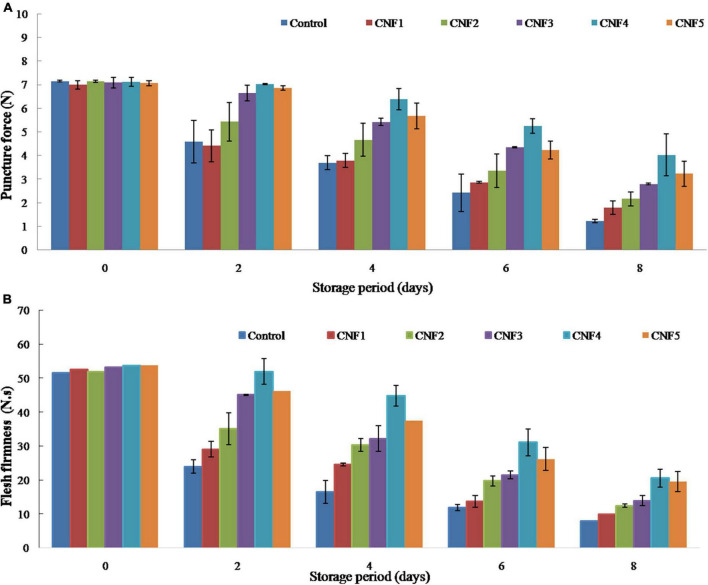
Puncture force **(A)** and flesh firmness **(B)** of sapota fruits stored in corrugated fibre board boxes containing CNF during storage at 26 ± 2°C.

Firmness is an important physical characteristic of fruit used to judge the freshness and market life of sapota fruit by consumers. Besides, high puncture force also indicates suitability for long-distance transport contributing valuably to high shelf life. The effect of different ethylene scavenging and antimicrobial nanofibres on puncture force (N) and flesh firmness of sapota fruit during storage at 26 ± 2°C for 8 days is shown in Figures 4A,B. While the puncture force in control fruits decreased by 82.9% after 8 days of storage, CNF fruits retained significantly more firmness. For CNF4 treatment, a 43.2% decrease was recorded. Further, the puncture force of fruits stored with CNF4 was 24–25% higher than those stored with CNF5, confirming the better performance of PdCl_2_ as compared to KMnO_4_ (Figure 2). The flesh firmness of sapota also displayed a linear decrease during storage. While fresh sapota showed firmness of 53.5 N.s, the 8 days stored sapota retained firmness of 19.6 N.s. While the presence of CNF in the package retained higher firmness during storage, the differences became less evident at the end of storage. On day 6, firmness greater than 25 N.s was recorded for both CNF4 and CNF5 treatments, with CNF4 recording higher firmness by 5 N.s. CNF4 treatment displayed 18.9% higher firmness compared to CNF5 on storage day 6. Higher firmness was recorded for tomatoes stored in the presence of KMnO_4_-loaded sepiolites by Hernandez et al. (16).

### Total soluble solids (°B)

**FIGURE 5:**
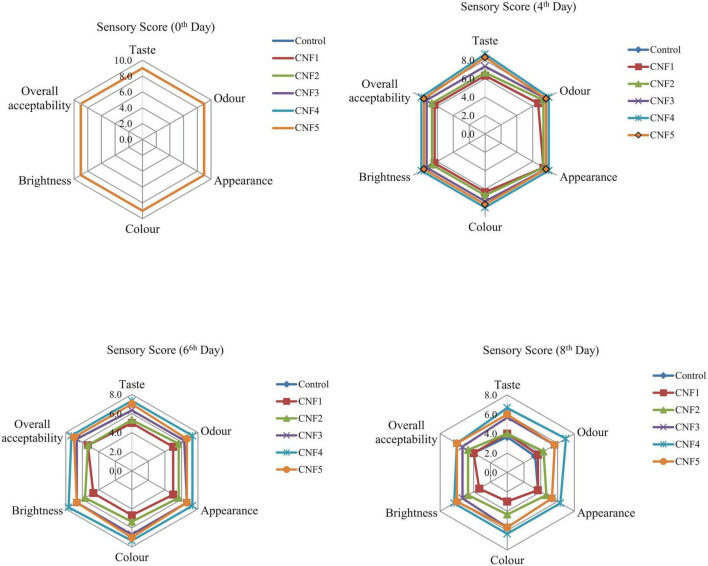
Sensory score of sapota fruits subjected to various nanomats treatment.

Total soluble solids content (TSS) of sapota fruit gradually increased after harvest with the progress of the storage period. Soluble solids level of 20% was found to be optimum in terms of palatability, as determined from the sensory score. A high increase in TSS is also undesirable during storage since it is linked to the moisture content of fruit tissue and the increase can be due to desiccation or ripening. On day 6 of storage, TSS values ranged in descending order are as follows: control (25.10°B) > CNF1 (25.00°B) > CNF2 (23.20°B) > CNF3 (23.50°B) > CNF4 (23.10°B) > CNF5 (23.77°B). The rate of increase in TSS of sapota fruit was delayed in the presence of CNF and among all the treatments, CNF4 showed a significantly lower increase of TSS (Table 3 and Figure 2). Even though the highest TSS of CNF3–CNF5 treatments was >23°B, it, remained lower by 2° over highest (25.1°B) in control. However, it maintained higher acceptability due to higher tissue firmness leading to the perception of freshness (Figure 5).

The rate of change in TSS for control, CNF1, CNF2, CNF3, CNF4, and CNF5 treatments during the 8 days storage period was 3.68, 3.66, 3.54, 3.6, 3.43, and 3.44%, respectively. Our findings are in agreement with the reports by Bhutia et al. (43) and Vijayalakshmi et al. (45) in case of CO 1, CO 2, and Kalipatti varieties of sapota subjected to ethylene absorbent treatment.

### Ascorbic acid

The ascorbic acid content of physiologically mature sapota fruits was 18 mg/100 g which progressively decreased to 6.8 mg/100 g at the end of storage on day 8 (Table 3). Our findings are in consonance with Tsomu et al. (46) who reported 12.40 mg/100 g ascorbic acid at the ripe stage in Sapota cv. Kalipatti. Ascorbic acid values for sapota fruit on day 6 of storage in descending order are as follows: CNF4 (15.33 mg/100 g) > CNF5 (15.00 mg/100 g) > CNF3 (14.33 mg/100 g) > CNF2 (10.45 mg/100 g) > CNF1 (9.89 mg/100 g) > control (9.78 mg/100 g). Progressive loss in ascorbic acid was observed across all the treatments and storage days but the rate of decrease varied according to the treatments (Figure 2). The decrease in ascorbic acid was rapid for control samples as compared to CNF4 samples. The rate of change in ascorbic acid for control, CNF1, CNF2, CNF3, CNF4, and CNF5 treatments during the 8 days storage period was 1.63, 1.69, 1.79, 2.19, 2.44, and 2.4%, respectively.

### Total phenolics and flavonoids

The sharp decline in the total phenolic content in control, CNF1 and CNF2 sample may be attributed to their faster desiccation and high respiration rate, which leads to the metabolic breakdown of stored phenols (47). Limited reports are available for phenolics content of sapota and explaining of sapota fruit during storage. In one study, Recuenco et al. (48) reported that the total phenolics content of sapota fruit at semi-ripe to ripe stages is 68 ± 4 mg GAE/100 g fw. Phenolic content may, however, vary according to the stage of fruit ripening, kind of varieties, and agroclimatic conditions. In the present study, the highest value of phenolic content in sapota fruit was 26.34 mg GAE/100 g FW.

Highest retention of total phenol content was noticed in CNF4 subjected samples (11.61 mg GAE/100 g), followed by CNF5 (9.90 mg GAE/100 g) and CNF3 (9.35 mg GAE/100 g) at the end of day 8 of storage (Table 3). Throughout the storage period, sapota fruits subjected to CNF4 and CNF5 treatment showed significantly (*p* < 0.05) higher retention of total phenolics as compared to control. On day 6, 34–37% of phenolics were retained by CNF4 and CNF5 treatment compared to fresh fruit. Compared to CNF5, 5–7% higher phenolics were recorded for fruits up to 6 days of storage for CNF4 fruits. This gap increased further on day 8 (17.27%) as CNF4 treatment could help retain higher phenolics during storage.

Along similar lines, in control, CNF1 and CNF2 samples, the total flavonoid content showed a sharp decline after day 2 of storage, reaching values of 2.66 mg/100 g in all three treatments. The maximum retention of total flavonoids was noticed in CNF4 samples (2.87 mg/100 g), followed by the CNF5 sample (2.83 mg/100 g) at the end of day 8 of storage (Table 3). Compared to fresh sapota, 58% of flavonoids were retained on day 6 of storage with CNF 4. CNF4 also showed 3.7–40% higher retention of flavonoids over CNF5 treatment during storage (Figure 2).

### Sensory properties

Sensory attributes of sapota fruits subjected to composite nanofibre treatment showed significant differences in taste, odour, colour, appearance, brightness, and overall acceptability as compared to control samples (*p* < 0.05) during storage at 26 ± 2°C for 8 days (Figure 5). During storage, the odour and taste (*p* < 0.05) of the fruit were significantly affected. The rate of deterioration was high in case of control samples as compared to nanofibre samples. Physiologically mature stage fruits showed an overall acceptability score of 9. The CNF4 and CNF5 nanofibre samples maintained higher sensory score at the end of day 8 of storage with overall acceptability scores in descending order as follows: control (4) < CNF1 (4) < CNF2 (4.7) < CNF3 (5.3) < CNF4 (6) ∼ CNF5 (6) on day 8 of storage (Figure 2). Off-odour in Control fruits was generally from anaerobic respiration and excessive ripening (49). The nanofibre samples maintained higher score with a mean sensory value of score = 5.2, as compared to control (Score = 4.0).

## Conclusion

The incorporation of ethylene scavengers in electrospun nanomats provides higher efficacy of active substances while overriding the use of free salts in sachets. Encapsulated palladium chloride proved to have higher efficacy in maintaining post-harvest shelf life of sapota compared to potassium permanganate as clearly shown in the heat map (Figure 2). Since ethylene plays a secondary role in the growth of fungi, such ethylene scavenging packaging aids with antimicrobial activity have immense potential to control fungal spoilage of subtropical climacteric horticultural commodities. The efficacy of composite nanofibres in enhancing the shelf life of highly climacteric sapota fruits consolidates its better effectiveness for other fruits. Humidity-triggered release of ethylene scavengers and essential oils from nanofibres can be further explored to design fruit package structures for subtropical climate conditions. These nanomats can have broad applications in the field of packaging and transportation of fruits, vegetables, and even flowers.

## Data availability statement

The original contributions presented in this study are included in the article/supplementary material, further inquiries can be directed to the corresponding author.

## Author contributions

GG: investigation, validation, and formal analysis. SR: conceptualization, writing—original draft, review and editing, and supervision. RP and TB: methodology, resources, and formal analysis. SS: visualization and supervision. SD: methodology and resources. AG: formal analysis. AA: visualization. All authors contributed to the article and approved the submitted version.
